# Systems Genetics Reveals the Gene Regulatory Mechanisms of *Arrb2* in the Development of Autism Spectrum Disorders

**DOI:** 10.3390/genes16050605

**Published:** 2025-05-20

**Authors:** Junyu Xia, Akhilesh K. Bajpai, Yamei Liu, Lele Yu, Yating Dong, Feng Li, Fuxue Chen, Lu Lu, Shini Feng

**Affiliations:** 1 School of Life Sciences, Shanghai University, Shanghai 200444, China; 2 Department of Genetics, Genomics and Informatics, University of Tennessee Health Science Center, Memphis, TN 38103, USA; 3 School of Environmental and Chemical Engineering, Shanghai University, Shanghai 200444, China; 4 Department of Laboratory Animal Science, Shanghai Public Health Clinical Center, Fudan University, Shanghai 201508, China

**Keywords:** autism spectrum disorder, *Arrb2*, systems genetics, gene regulation, BXD mice, candidate gene, QTL mapping

## Abstract

Background: Autism spectrum disorder (ASD) involves complex interactions between genetic and environmental factors. Recent studies suggest that dysregulation of β-arrestin2 (*Arrb2*) in the central nervous system is linked to ASD. However, its specific mechanisms remain unknown. Methods: This study employs a systems genetics approach to comprehensively investigate *Arrb2* in multiple brain tissues, including the amygdala, cerebellum, hippocampus, and prefrontal cortex, using BXD recombinant inbred (RI) strains. In addition, genetic variance analysis, correlation analysis, expression quantitative trait loci (eQTL) mapping, and functional annotation were used to identify the key downstream targets of *Arrb2*, validated by quantitative reverse transcription polymerase chain reaction (qRT-PCR) and Western blotting (WB). Results: *Arrb2* exhibited expression variations across the four brain regions in BXD mice. eQTL mapping revealed that *Arrb2* is cis-regulated, and increased *Arrb2* expression levels were significantly correlated with ASD-like symptoms, such as impaired social interactions and abnormal learning and memory. Furthermore, protein–protein interaction (PPI) network analysis, tissue correlation, functional relevance to autism, and differential expression identified eight downstream candidate genes regulated by *Arrb2*. The experimental results demonstrated that deletion of *Arrb2* led to the downregulation of *Myh9*, *Dnmt1*, and *Brd4* expression, along with protein kinase A (PKA)-induced hyperactivation of Synapsin I. These findings suggest that *Arrb2* may contribute to the pathogenesis of autism by modulating the expression of these genes. Conclusions: This study highlights the role of *Arrb2* in ASD pathogenesis and identifies *Myh9*, *Dnmt1*, and *Brd4* as key downstream regulators. These findings provide new insights into the molecular mechanisms of ASD and pave the way for novel therapeutic targets.

## 1. Introduction

β-arrestin2 (*Arrb2*), a multifunctional adaptor protein belonging to the β-arrestin (Arrb) family, is widely distributed across the cytoplasm, nucleus, and plasma membrane [1]. In the classical G-protein signaling pathway, *Arrb2* directly inhibits GPCR-mediated signaling by competing with G proteins for binding to G-protein-coupled receptors (GPCRs). Beyond its canonical functions in GPCR desensitization, this adaptor protein coordinates regulatory functions encompassing internalization and transport [2]. Moreover, as a multifunctional scaffold for several signal transduction proteins, *Arrb2* can integrate and regulate various signaling pathways, including Wnt, TGF-β, MAPK, and AMPK/mTOR, making it a potential therapeutic target for multisystem diseases [3].

*Arrb2* is closely associated with the development of several neurodegenerative diseases, such as Parkinson’s disease (PD), frontotemporal dementia (FTD), and Alzheimer’s disease (AD) [4]. However, studies linking this gene with Autism spectrum disorder (ASD) are limited. According to earlier studies conducted in our lab, a mouse model of autism generated by valproic acid (VPA) showed a substantial increase in *Arrb2* expression [5]. Furthermore, *Arrb2*^−/−^ mice exhibit higher electroencephalogram (EEG) rhythms [6], mitochondrial dysfunction, and downregulated autophagy in hippocampal neurons [7]. These studies imply that *Arrb2* may serve as a critical factor in autism development, but the specific genetic mechanisms and molecular networks of *Arrb2* in brain tissues warrant further investigation.

ASD is a neurodevelopmental disorder [8], characterized by core symptoms, such as abnormal communication deficits, social interactions, and repetitive behaviors [9]. In addition, patients often experience a variety of comorbidities, such as intellectual disability, epilepsy, anxiety, gastrointestinal issues, and sleep problems [10]. As the pathogenesis of autism is not fully understood and is significantly heterogeneous, current clinical diagnosis relies mainly on abnormal behavioral manifestations rather than specific biomarkers, posing significant challenges to accurate diagnosis [11]. Therefore, it is imperative to identify better diagnostic/prognostic/therapeutic targets for autism.

Recent studies have revealed that autism is closely related to structural and functional abnormalities in multiple brain tissues. The amygdala is a well-known emotional processing structure. Functional magnetic resonance imaging (fMRI) studies reveal that when autistic children are exposed to varied facial expressions, their amygdala activation levels are often lower than those of the control group, impairing their emotional recognition abilities [12]. A brain imaging study focusing on the cerebellum has shown that autistic patients exhibit volume abnormalities in cerebellar sub-regions, especially in the right lobule VI and vermis VI-VII, which are closely related to social cognition, memory, and emotion regulation functions [13]. The hippocampus is vital for memory and spatial navigation. Multiple studies have found that autism is associated with abnormal neurodevelopment in the hippocampus, particularly in neural connectivity [14], neuronal proliferation [15,16], and synaptic plasticity [17,18]. The prefrontal cortex is critical for high-level cognitive activities and decision-making. In autistic patients, changes in the number of excitatory and inhibitory synapses may lead to neuronal dysfunction and network connectivity disorders in the prefrontal cortex, impairing decision-making and social interactions [19]. In summary, investigating the mechanisms of autism in multiple brain regions may provide important clues for understanding its neurobiological basis and laying the foundation for future intervention and treatment strategies.

Systems genetics is a comprehensive field of research that relies on genetic reference population (GRP), emphasizes multi-level data integration and analysis, and aims to elucidate the genetic basis of complex traits and their biological mechanisms [20]. The BXD family, a recombinant-inbred strain derived from the crosses between C57BL/6J and DBA/2J mice is currently one of the most widely used mouse GRP globally [21]. Currently, this family contains more than 150 inbred strains separated for approximately 6 million single-nucleotide variants and have been phenotyped for over 10,000 different physiological and behavioral traits [22,23]. The rich data and consistent genetic background of BXD mice have enabled their extensive application in studies related to understanding the genetic mechanisms of neurodegenerative diseases [24,25,26], cardiovascular diseases [27,28,29], and visual system degeneration [30,31,32]. Recent studies using the BXD mice have revealed variable penetrance of the autism-risk gene *Chd8* in mediating complex behavioral phenotypes, including cognitive impairment and social deficits [33]. Furthermore, several studies have characterized the genetic architecture underlying ASD-associated phenotypes, such as atypical infant vocalizations [34], and corpus callosum dysplasia [35,36]. Our investigation focuses on *Arrb2*, an emerging autism-risk candidate gene, to systematically dissect its pivotal role in orchestrating multi-brain-region gene regulatory networks and modulating autism pathogenesis.

In this study, we used a systems genetics approach to explore the role of *Arrb2* in autism. To this end, we evaluated the expression variation in *Arrb2* in four brain regions (amygdala, cerebellum, hippocampus, and prefrontal cortex) of BXD mice and its correlation with autism-related traits. Furthermore, *Arrb2*-correlated genes were used to explore potential signaling pathways, and subsequently identify downstream regulators that might be involved in the development of autism. Finally, the downstream regulators were experimentally validated in knock-out mice models.

## 2. Materials and Methods

### 2.1. Arrb2 Expression Data in BXD Family

*Arrb2* is expressed in multiple brain regions, including the amygdala, cerebellum, hippocampus, and prefrontal cortex. The expression data for these brain regions of BXD mice were generated by us and our collaborators using microarray and are publicly accessible through our GeneNetwork version 2 (v2) platform (http://genenetwork.org/) [37].

The amygdala dataset (GN280: INIA Amygdala Affy MoGene 1.0 ST (Nov10) RMA) was generated from F1 hybrids (B6D2F1 and D2B6F1), parental strains (C57BL/6J and DBA/2J), and 54 BXD recombinant inbred (RI) strains. The cerebellum dataset (GN56: SJUT Cerebellum RNA M430 (Mar05) RMA) [38] comprised of 45 BXD RI strains, two parental strains, and F1 hybrids, with gene expression estimates averaged across D2B6F1 and B6D2F1 hybrids. The hippocampus dataset (GN110: Hippocampus Consortium M430v2 (Jun06) RMA) [39] included 67 BXD RI strains, parental strains, and F1 hybrid strains. The prefrontal cortex mRNA expression dataset was generated from 27 BXD RI strains and the parental strains (GN135: VCU BXD PFC Sal M430 2.0 (Dec06) RMA) [40]. All animals were sacrificed during the light phase, which lasted from 9:00 AM to 5:00 PM. RNA extraction was performed using the RNA STAT-60 technique, which includes tissue homogenization, RNA isolation, precipitation, and washing. Purification was completed with NaOAc before microarray profiling. Additional details on the RNA extraction protocols can be found in our GeneNetwork database by accessing the individual datasets. For example, the amygdala dataset can be accessed by selecting Species as “Mouse”, Group as “BXD Family”, Type as “Amygdala mRNA” and Dataset as “INIA Amygdala Affy MoGene 1.0 ST (Nov10) RMA”.

### 2.2. Microarray Analysis and Data Processing

Raw microarray data files were processed with the Robust Multi-Chip Average (RMA) method [41], followed by log2 transformation and Z-score normalization as described previously [39]. To eliminate negative values, the normalized distribution was adjusted to a mean of 8 and a standard deviation of 2 using the 2Z + 8 transformation.

### 2.3. Correlation Analysis

Gene–gene and gene–trait correlations were analyzed using Pearson correlation coefficients. For gene–gene correlation analysis, *Arrb2* mRNA expression from four brain regions (amygdala, cerebellum, hippocampus, and prefrontal cortex) of the BXD mice was compared with the expression of other genes in the same dataset. For gene–trait correlation analysis, potential associations between *Arrb2* expression in each brain region and 424 ASD-related traits were assessed. ASD-related traits were selected based on a systematic review [42], which cataloged animal models for ASD research. We used the keywords from this review to batch-query and retrieved matching phenotypes from all published BXD datasets in GeneNetwork v2.

### 2.4. Expression Quantitative Trait Loci (eQTL) Mapping

The webQTL module in GeneNetwork was used to perform eQTL mapping for *Arrb2* expression variation in BXD mice [43]. This analysis used the Genome-wide Efficient Mixed-Model Association (GEMMA) method to calculate *p*-values, with a linear mixed model to account for sample relatedness [44]. To avoid overcorrection of nearby genetic variants, the Leave-One-Chromosome-Out (LOCO) method was applied, and association results were reported as −log10(*p*). The suggestive and significant thresholds for the genome-wide scan were −log10(*p*) of 2.5 and 4.0, respectively.

### 2.5. Gene Enrichment Analysis

Using the Web-based GEne SeT Analysis Toolkit (WebGestalt v2019, http://www.webgestalt.org, accessed on 14 January 2024) [45], genes that were significantly correlated with *Arrb2* (|*r*| ≥ 0.3, *p* < 0.05, and average expression level ≥ 7.1) were functionally analyzed. The enrichment of Kyoto Encyclopedia of Genes and Genomes (KEGG) pathways and Mammalian Phenotype Ontology (MPO) annotations were analyzed using default parameters. Protein-coding genes as the reference set and a minimum of 5 genes per category were selected as parameters for the enrichment analysis. A False Discovery Rate (FDR) < 0.1 was considered as a significance threshold.

### 2.6. Protein–Protein Interaction (PPI) Network

The Search Tool for the Retrieval of Interaction Gene/Proteins (STRING v12, https://cn.string-db.org/, accessed on 28 January 2024) [46] database was used to construct a PPI network using *Arrb2*-correlated genes, aiming to identify key genes interacting with *Arrb2*. The correlated genes were submitted as a list under the “Multiple proteins” option, and “Mus musculus” was selected as the organism. The degree value—an indicator reflecting the number of direct protein–protein interactions—was analyzed using the cytoHubba plugin in Cytoscape v3.10.1 [47].

### 2.7. Differential Expression Between Human Autism and Control Samples

To investigate the differentially expressed genes (DEGs) between human autism and controls, we analyzed publicly available gene expression datasets across three distinct tissues: cerebellum, prefrontal cortex, and peripheral blood. GSE38322 [48,49] included eight autism and eight control cerebellar samples, as well as six autism and four control BA19 samples. GSE113834 [50] included 15 male autism patients and 12 controls (prefrontal cortex BA8/9), while GSE26415 [51] compared 21 young individuals with autism and 21 healthy mothers of autistic children (peripheral blood).

All data were downloaded from the NCBI-GEO database (https://www.ncbi.nlm.nih.gov/geo, accessed on 17 January 2024), and then background-corrected and normalized using RMA in R v4.3.2 [52], and limma v3.58.1 [53] was used for differential expression analysis. Differentially expressed genes were considered based on FDR-corrected (Benjamini–Hochberg method) *p* < 0.1.

### 2.8. Candidate Gene Selection

To rank the genes and further select the potential candidate(s), primary and secondary interactors of *Arrb2* were considered for scoring. The scoring system consists of four weighted parameters ranging from 0 to 5. The parameters were based on the data obtained from BXD mice as well as functional resources as follows: (1) PPI network node degree: Genes with a node degree > 1 (interacting with >1 protein in the PPI network) received a score of 1; (2) Tissue-correlation analysis: Genes significantly (*p* < 0.05) correlated with *Arrb2* in any of the four brain regions (amygdala, cerebellum, hippocampus, prefrontal cortex) were assigned a score of 2; (3) Functional relevance to autism: Genes linked to autism were identified using the keyword “Autism” from rodent databases, Mouse Genome Informatics (MGI, http://www.informatics.jax.org, accessed on 16 January 2024) [54] and Rat Genome Database (RGD, https://rgd.mcw.edu, accessed on 16 January 2024) [55], and human databases, GeneCards v5.19 (https://www.genecards.org, accessed on 16 January 2024) [56] and AutDB (http://autism.mindspec.org/autdb, accessed on 16 January 2024) [57]. These lists of genes were then matched with *Arrb2*-interactors, and those overlapping were assigned a score of 1; (4) Differential expression: Genes significantly differentially expressed between autism patients and control samples (in cerebellum, prefrontal cortex, or peripheral blood) were assigned a score of 1. Finally, genes with a total score ≥ 4 (80% of maximum) were selected as downstream targets of *Arrb2* that may modulate autistic traits.

### 2.9. Phenome-Wide Association Study (PheWAS)

Potential phenotypes linked to genetic variants can be found using the reverse genetic analysis technique PheWAS v1 [58]. In this study, we estimated the relationship between each gene and clinical features by analyzing roughly 5000 clinical phenotypes and using the multi-locus mixed-model technique (mlmm). This analysis was performed on the SystemsGenetics platform at EPFL (https://systems-genetics.org, accessed on 28 January 2024).

### 2.10. Animals

The experimental animals were adult C57BL/6 mice (Shanghai Laboratory Animal Center of the Chinese Academy of Sciences) and *Arrb2*^−/−^ mice (Shanghai Public Health Clinical Center). Mice were randomly selected from their respective genotype cohorts. The animal experiment protocol closely adhered to the applicable rules set by the Animal Ethics Committee of Shanghai University (ECSHU-2020-030). All mice were accommodated in an animal facility free of specific pathogens on a conventional 12 h light/12 h dark cycle, with sterilized drinking water and diet provided.

### 2.11. Quantitative Real-Time Polymerase Chain Reaction (qRT-PCR)

Following the manufacturer’s instructions, total RNA was extracted from the hippocampus of mice using the EasyPure RNA Kit (TransGen Biotech, ER101-01, Beijing, China). Utilizing the cDNA synthesis kit (Yeasen Biotech, 11141ES10, Shanghai, China), RNA was reverse-transcribed into cDNA. To standardize the relative expression changes in the genes, the expression of the internal reference gene *Gapdh* was calculated using the SYBR Green detection system (Bio-Rad, CA, USA) for qRT-PCR techniques. The primers for target genes are listed in Table 1.

### 2.12. Western Blotting

Hippocampal proteins were extracted using a denaturing lysis buffer supplemented with protease inhibitors. Protein lysates were quantified via BCA assay, and equal masses were resolved on a 4–20% SDS-PAGE gel. Following electrophoresis, proteins were transferred to a PVDF membrane and blocked with 5% non-fat milk for 90 min. The PVDF membrane was incubated at 4 °C overnight with primary antibodies, including mouse anti GAPDH (HRP-conjugated, 1:10,000, Proteintech, 60,004, Wuhan, Hubei, China), rabbit anti β-Arrestin2 (1:1000, Cell Signaling Technology, 3857, Danvers, Massachusetts, USA), rabbit anti Dnmt1 (1:2000, HuaBio, ET1702-77, Hangzhou, Zhejiang, China), rabbit anti Myh9 (1:2000, Abclonal, A0173, Wuhan, Hubei, China), rabbit anti Brd4 (1:1000, HuaBio, HA722785, Hangzhou, Zhejiang, China), rabbit anti p-Synapsin I (S9) (1:1000, HuaBio, ET1611-26, Hangzhou, Zhejiang, China), rabbit anti Synapsin I (1:1000, abcam, ab64581, Cambridge, UK) and rabbit anti p-PKA α/β/γ (catalytic subunit) (T197) (1:1000, HuaBio, HA721864, Hangzhou, Zhejiang, China). After three 10-min PBST washes, membranes were probed with an HRP-conjugated goat anti-rabbit IgG secondary antibody (1:5000, ABclonal, AS014, Wuhan, Hubei, China) at room temperature for 90 min. Protein bands were visualized using an enhanced ECL chemiluminescence kit (Yeasen, 36208ES60, Shanghai, China), and the band intensities were quantified using ImageJ software. v1.54d.

### 2.13. Statistical Analysis

All statistical analyses were performed using GraphPad Prism software v8.0.2. Data are presented as mean ± SD. Intergroup comparisons were performed using unpaired two-tailed Student’s *t*-tests, with statistical significance thresholds defined as * *p* < 0.05, ** *p* < 0.01, and *** *p* < 0.001.

## 3. Results

### 3.1. Expression Variability of Arrb2 in the BXD Family and Its Association with Autism Phenotypes

To investigate the feasibility of using systems genetics approaches and BXD mice to study the biological function of *Arrb2*, we analyzed its expression in the amygdala, cerebellum, hippocampus, and prefrontal cortex of the BXD family. The transcriptome data of BXD RI mice were obtained from our GeneNetwork database.

**Figure 1:**
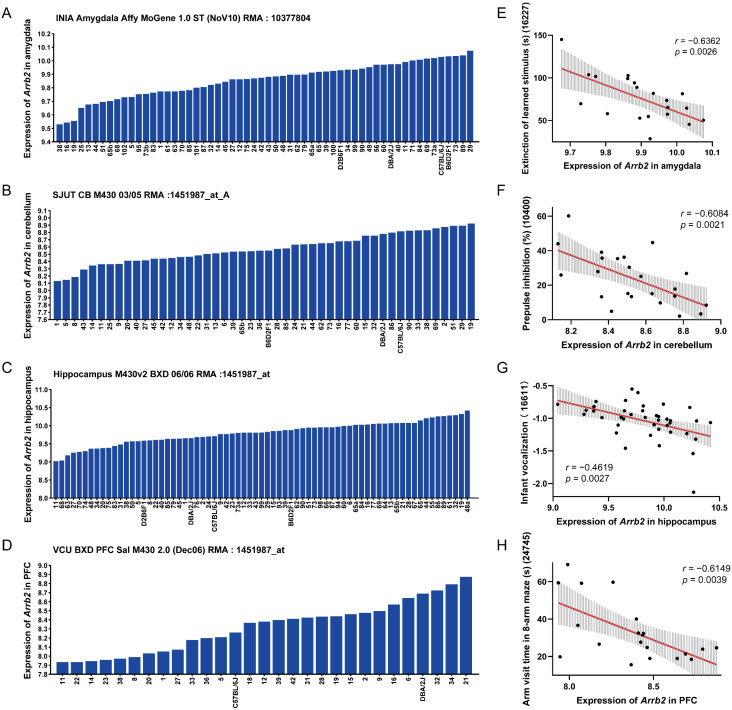
*Arrb2* expression in the BXD family and its association with autism phenotypes. Bar charts show the *Arrb2* expression levels in the (**A**) amygdala, (**B**) cerebellum, (**C**) hippocampus, and (**D**) prefrontal cortex of the BXD family. The *x*-axis represents BXD strains, parental strains, and F1 hybrids, while the *y*-axis represents the normalized log2 expression levels of *Arrb2*. Scatter plots show the correlations between *Arrb2* expression levels in the four brain regions and (**E**) extinction of learned stimulus (Record ID 16227), (**F**) prepulse inhibition (Record ID 10400), (**G**) infant vocalization (Record ID 16611), and (**H**) arm visit time in the eight-arm maze (Record ID 24745), respectively. Pearson correlation coefficient *r* and *p*-values are indicated. The red solid line shows the linear regression fit, and the gray shaded area indicates the 95% confidence interval of the regression line, reflecting the uncertainty of the fit.

In the amygdala of 58 BXD strains, *Arrb2* expression was 9.856 ± 0.131 SD, showing a 1.459-fold difference (Figure 1A). In the cerebellum, the average *Arrb2* expression across 48 BXD strains was 8.576 ± 0.203 SD, with a 1.731-fold difference (Figure 1B). In the hippocampus of 67 BXD strains, the expression value was 9.802 ± 0.313 SD, demonstrating a 2.641-fold difference (Figure 1C). In the prefrontal cortex, the average expression in all 29 BXD strains was 8.321 ± 0.28 SD, with a 1.919-fold difference (Figure 1D). These results indicate significant expression variability of *Arrb2* across multiple brain regions in the BXD family. Therefore, we considered these strains for subsequent studies on detailed pathophysiology and functional roles of *Arrb2*.

Additionally, we analyzed the correlation between *Arrb2* expression in each brain region and 424 autism-related phenotypes selected from GeneNetwork, based on a review article [42]. Using the Pearson correlation method, we identified significant correlations between *Arrb2* expression and specific autism phenotypes (Supplementary File S1). The phenotypes most significantly correlated with *Arrb2* expression in the amygdala, cerebellum, hippocampus, and prefrontal cortex were as follows: extinction of learned stimulus using a touch screen [59] (*r* = −0.6362, *p* = 0.0026, Figure 1E), prepulse inhibition of the acoustic startle reflex [60,61,62] (*r* = −0.6084, *p* = 0.0021, Figure 1F), infant vocalization [34] (*r* = −0.4619, *p* = 0.0027, Figure 1G), and eight-arm maze visit time [63] (*r* = −0.6149, *p* = 0.0039, Figure 1H), respectively. These results suggest that higher *Arrb2* expression is negatively associated with core autism symptoms, potentially impairing learning and memory, sensorimotor gating, and language development in the BXD strains.

### 3.2. eQTL Mapping Reveals That Arrb2 Expression in the Mouse Brain Is Cis-Regulated

**Figure 2:**
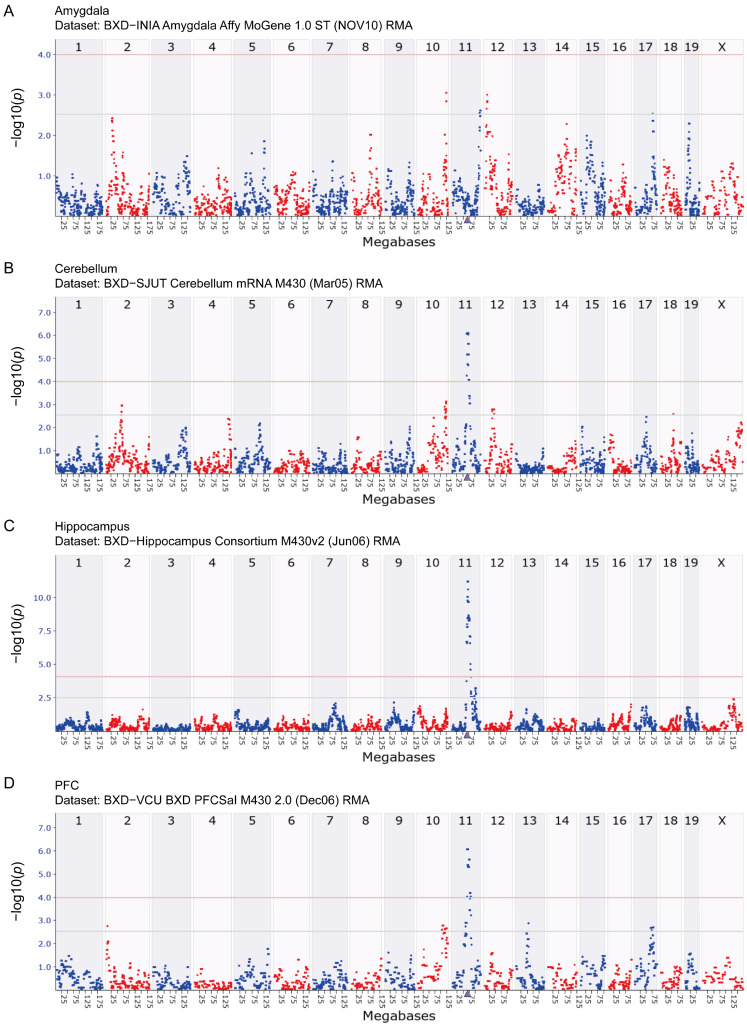
eQTL mapping of *Arrb2* in BXD mice. Manhattan plots depict the chromosomal loci regulating *Arrb2* expression in the (**A**) amygdala, (**B**) cerebellum, (**C**) hippocampus, and (**D**) prefrontal cortex of the BXD family. The *x*-axis represents the mouse genome position in megabases (Mb), and the *y*-axis shows −log10(*p*), quantifying associations between *Arrb2* expression and genomic loci. The purple triangle marks the genomic location of *Arrb2*. Grey and red horizontal lines denote genome-wide suggestive (−log10(*p*) = 2.5) and significant (−log10(*p*) = 4.0) thresholds, respectively. The differentiation between red and blue scatter points is aimed at clearly distinguishing between the scatter points of adjacent regions and does not represent any actual data differences.

The results of eQTL mapping provide critical insights into the regulatory mechanisms governing *Arrb2* expression. Using the GEMMA method, no significant amygdala eQTL was identified (Figure 2A), whereas significant eQTLs were observed in the cerebellum, hippocampus, and prefrontal cortex. For the cerebellum, a significant eQTL was located within the 63.6–75.4 Mb on chromosome 11 (Figure 2B). For the hippocampus, the genomic region associated with *Arrb2* expression was mapped to 64.4–80.6 Mb on chromosome 11 (Figure 2C). For the prefrontal cortex, a significant eQTL was detected in the 64.4–78.2 Mb of chromosome 11 (Figure 2D).

These eQTLs overlapped with or neighbored the *Arrb2* locus (Chr11: 70.439877 Mb), indicating that they are cis-eQTLs. This suggests that sequence variations in cis-regulatory elements within or near *Arrb2* directly influence its expression. Consequently, we propose to investigate *Arrb2*’s potential downstream targets, which may exert genetic regulation through transcript-level expression modulation.

### 3.3. Arrb2-Correlated Genes Are Enriched in Neural Pathways and Phenotypes

Genes typically exert their functions through interactions with other genes, either directly or indirectly. Consequently, functionally related gene sets are often co-regulated in specific tissues or cell types. To investigate *Arrb2*’s biological roles in four brain regions (amygdala, cerebellum, hippocampus, and prefrontal cortex), we conducted genetic correlation analyses between *Arrb2* and other genes in the transcriptomes of these brain regions. We identified 6,634, 1,736, 2,941, and 906 genes significantly correlated with *Arrb2* expression in the amygdala, cerebellum, hippocampus, and prefrontal cortex, respectively (|*r*| ≥ 0.3, *p* < 0.05; mean expression ≥ 7.1). The complete gene list is provided in Supplementary File S2. Pathway and phenotype enrichment analyses of these genes revealed potential mechanisms underlying *Arrb2*’s functional roles in BXD mice.

**Figure 3:**
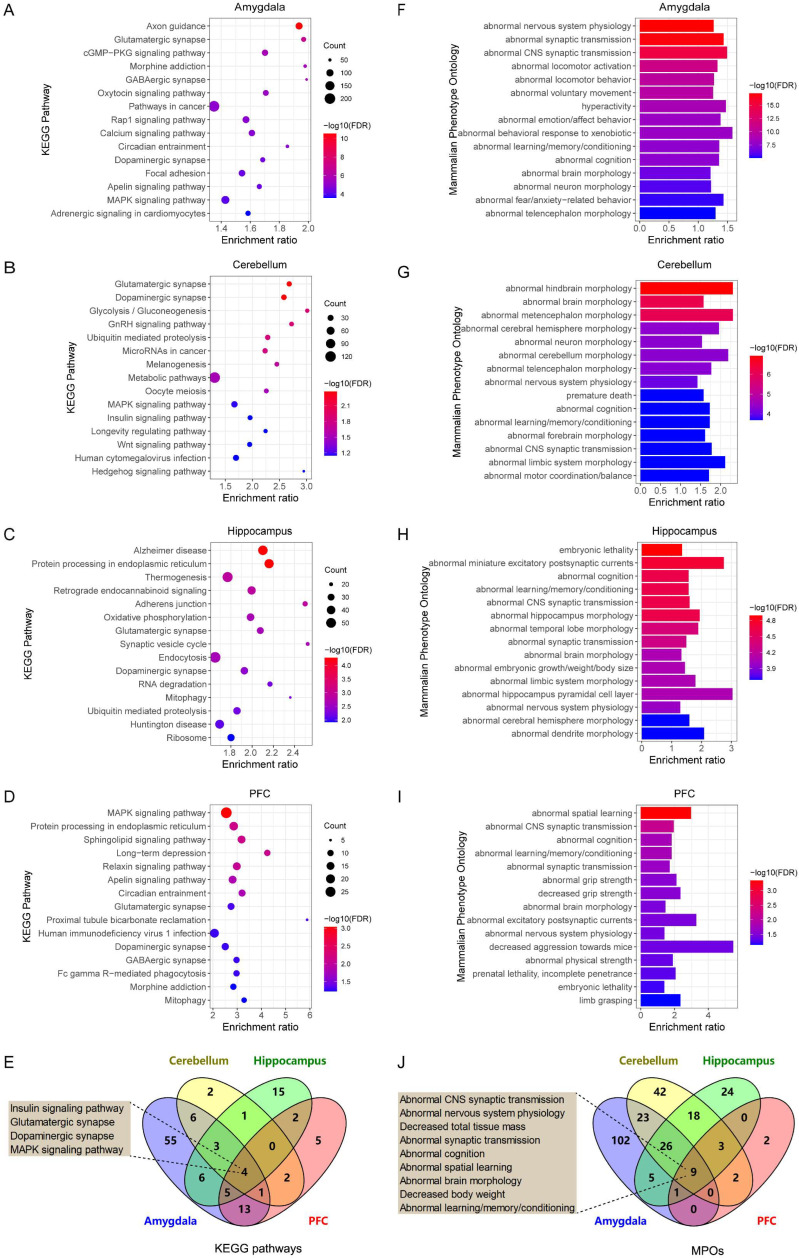
Functional enrichment analysis of *Arrb2*-correlated genes. Bubble plots depict the top 15 KEGG pathways significantly enriched by *Arrb2*-correlated genes in the (**A**) amygdala, (**B**) cerebellum, (**C**) hippocampus, and (**D**) prefrontal cortex of BXD mice (FDR < 0.1), along with (**E**) the shared KEGG pathways among the four brain regions. Additionally, bar charts show the top 15 mammalian phenotype Ontologies (MPOs) significantly enriched by *Arrb2*-correlated genes in the (**F**) amygdala, (**G**) cerebellum, (**H**) hippocampus, and (**I**) prefrontal cortex of BXD mice (FDR < 0.1), and (**J**) the common MPOs among the four brain regions. In the bubble plots and bar charts, the *x*-axis represents the enrichment ratio, while the *y*-axis represents the enriched pathways or terms. The size of each dot corresponds to the number of genes, and the color indicates the *p*-value. The boxes beside the Venn diagrams display the pathways or MPOs enriched in all four brain regions.

The top 15 KEGG pathways enriched by *Arrb2*-correlated genes in each brain region are presented as bubble plots (Figure 3A–D). Furthermore, we identified four KEGG pathways significantly enriched across all four brain regions (Figure 3E). Among these, the “insulin signaling pathway” plays a role in cognitive and memory functions by modulating neuronal metabolism, structure, and neurotransmitter transmission [64]. The “glutamatergic synapses” and “dopaminergic synapses” pathways are associated with neurological and mental disorders, including Parkinson’s disease and schizophrenia [65]. Additionally, abnormal synaptic function is recognized as a key factor in autism development [66]. Another interesting pathway is the “MAPK signaling pathway” [67], which offers insights into the intracellular signal transduction of *Arrb2* and the regulation of downstream genes.

We also performed MPO enrichment analysis of *Arrb2*-correlated genes in each brain region. The top 15 abnormal phenotypes in each brain region are depicted as bar charts (Figure 3F–I). Comparing the enriched MPO terms revealed nine overlapping phenotypes across all four brain regions (Figure 3J), and most of them were related to nervous system functions, such as “abnormal synaptic transmission”, “abnormal cognition”, and “abnormal learning/memory/conditioning”. The complete lists of significantly enriched KEGG pathways and MPOs are provided in Supplementary File S3.

### 3.4. PPI Network Analysis Infers Key Downstream Targets of Arrb2

**Figure 4:**
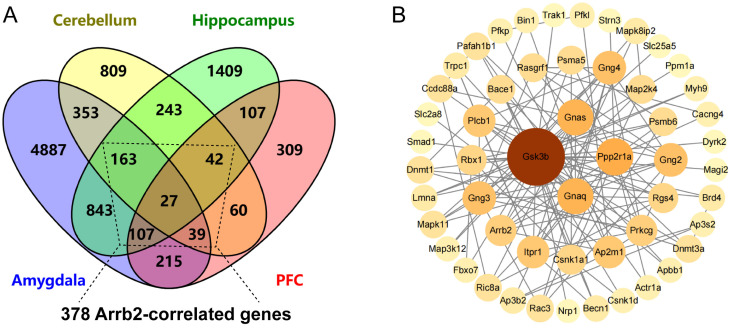
Inferring key downstream molecules of *Arrb2*-correlated genes through protein–protein interaction (PPI) network analysis. (**A**) Venn diagram showing the overlap of *Arrb2*-correlated genes across four brain regions (amygdala, cerebellum, hippocampus, prefrontal cortex) in BXD mice. (**B**) PPI network nodes representing proteins derived from the mapping of their corresponding genes Node color intensity and diameter are proportional to the degree value.

To further identify the key downstream targets among *Arrb2*-correlated genes, we submitted 378 *Arrb2*-correlated genes expressed in at least three of four brain regions (amygdala, cerebellum, hippocampus, and prefrontal cortex) (Figure 4A) using the STRING database, where they were mapped to their corresponding proteins for PPI analysis. The PPI network showed 53 proteins that are primary and secondary interactors of Arrb2 (Figure 4B).Notably, seven proteins (Gsk3b, Ppp2r1a, Gnaq, Gnas, Ap2m1, Rgs4, Map2k4) directly interacted with Arrb2 occupied the central part of the network and exhibited high connectivity (degree ≥ 5). Detailed information for all 53 genes is provided in Supplementary File S4.

### 3.5. Differentially Expressed Genes Between Human Autism and Control Samples

To further narrow down the *Arrb2*-interactors, we utilized the clinical data collected from human samples to identify differentially expressed genes between autism patients and controls.

**Figure 5:**
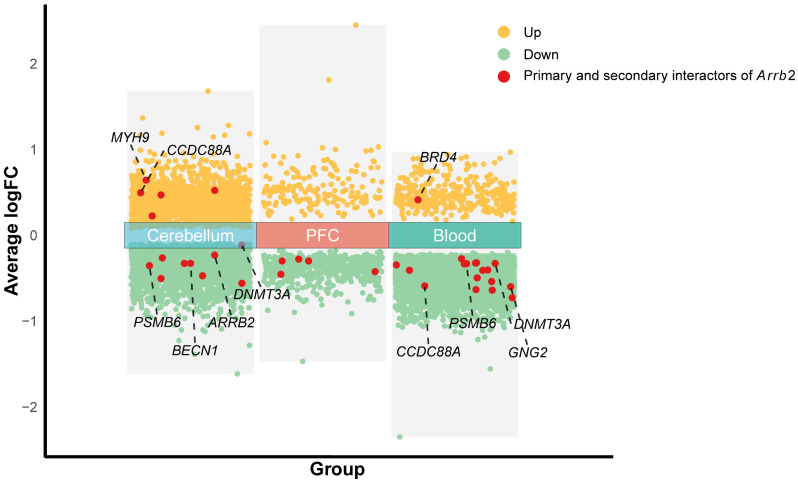
Volcano plot of genes differentially expressed between human autism and control samples. The *x*-axis displays group labels, while the *y*-axis indicates the average log fold change (logFC) values. Yellow scatter points represent genes upregulated significantly (FDR-adjusted *p*-value < 0.1), green scatter points denote significantly downregulated genes, and red scatter points highlight genes that are primary or secondary interactors of *Arrb2*.

Using publicly available gene expression microarray data from the NCBI-GEO database, we identified genes with differential expression between human autism and control samples in three tissues: the cerebellum (GSE38322), prefrontal cortex (GSE113834), and peripheral blood (GSE26415) (Figure 5). The complete list of significantly differentially expressed genes is provided in Supplementary File S5. Figure 5 shows genes that overlap between BXD mice and humans. Investigating the expression patterns of these shared genes in mouse models is critically important for a better understanding of human diseases.

### 3.6. Identification of Strong Candidate Genes Regulated by Arrb2

To prioritize candidates, a composite scoring system (0–5 points) was applied, integrating evidence from GeneNetwork and multi-species public databases. Within the BXD genetic reference population, 53 genes exhibiting functional interactions with *Arrb2* (primary or secondary) were identified, each demonstrating a network connectivity degree > 1 in the PPI analysis. Among these, 10 genes displayed a tissue-correlation with *p* < 0.05 in any of the four brain regions (amygdala, cerebellum, hippocampus, and prefrontal cortex), suggesting region-dependent transcriptional regulation. Then, functional enrichment analysis across human, murine, and rat databases revealed that 19 genes were linked to autism. Concurrently, 28 genes exhibited differential expression (FDR < 0.1) between ASD and control samples in the cerebellum, prefrontal cortex, or peripheral blood transcriptomic datasets.

Consequently, eight genes (*Dnmt3a*, *Myh9*, *Ccdc88a*, *Dnmt1*, *Becn1*, *Gng2*, *Psmb6*, *Brd4*) achieved scores exceeding four (representing 80% of maximal weight) and were thereby defined as strong *Arrb2*-regulated candidates (Table 2).

### 3.7. PheWAS Analysis of Genetic Variations in Candidate Genes

**Figure 6:**
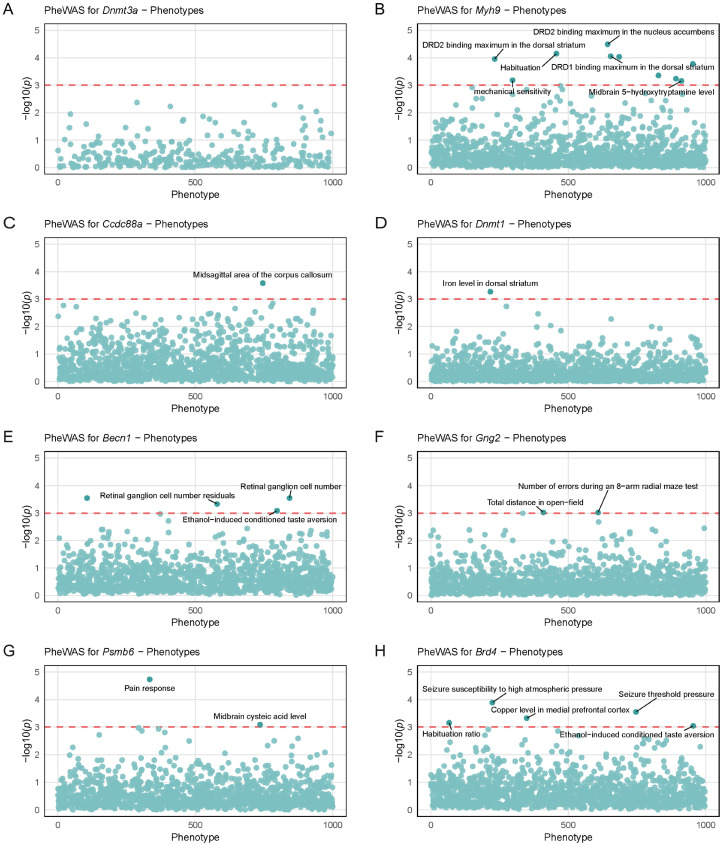
Phenome-wide association study (PheWAS) analysis of genetic variations in strong candidate genes regulated by *Arrb2*. Scatter plots show the PheWAS results for (**A**) *Dnmt3a*, (**B**) *Myh9*, (**C**) *Ccdc88a*, (**D**) *Dnmt1*, (**E**) *Becn1*, (**F**) *Gng2*, (**G**) *Psmb6*, and (**H**) *Brd4*. Each scatter represents a phenotype, and the *x*-axis coordinates of the scatter are randomly distributed. The *y*-axis represents the association significance between each gene and central nervous system-related traits in BXD mice, with the red dotted line marking the statistical significance threshold (–log10(*p*) = 3).

PheWAS is a reverse genetic analysis approach. Using eight strong candidate genes regulated by *Arrb2* and approximately 5000 clinical phenotypes from BXD RI strains, we identified potential phenotypes associated with genetic variations in these candidates. A q-value (i.e., FDR-adjusted *p*-value) < 0.05 was considered statistically significant. To rigorously control false positive discoveries and enhance result reliability, we implemented a more stringent significance threshold (*q* < 0.001). In BXD mice, *Dnmt3a* showed weak associations with central nervous system (CNS) traits, with no phenotypes surpassing the *q* < 0.001 threshold (Figure 6A). The other seven candidate genes exhibited strong associations with CNS traits (Figure 6B–H), primarily involving neurotransmitter receptor-binding capacity, neurotransmitter levels, habituation ability, brain structural features, behavioral tests, seizure susceptibility, and conditioned aversion. These traits collectively reflect diverse functional and regulatory mechanisms of the nervous system.

### 3.8. Knocking Out Arrb2 Downregulates the Expression of Downstream Candidate Genes in the Hippocampus of Mice

**Figure 7:**
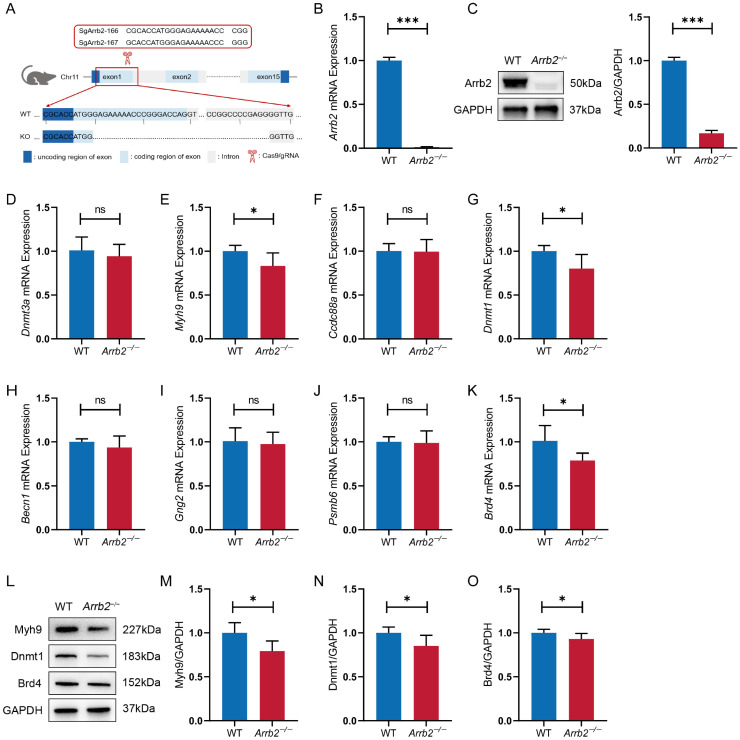
Construction of *Arrb2*^−/−^ mice and analysis of mRNA and protein levels of downstream candidate genes in the hippocampus. (**A**) Schematic diagram of the construction of *Arrb2*^−/−^ mice. (**B**) qRT-PCR analysis of *Arrb2* mRNA in the hippocampus of WT and *Arrb2*^−/−^ mice. (**C**) Western blot analysis of Arrb2 protein in the hippocampus of WT and *Arrb2*^−/−^ mice. (**D**–**K**) mRNA levels of downstream candidate genes regulated by *Arrb2* in the hippocampus of WT and *Arrb2*^−/−^ mice measured by qRT-PCR. (**L**–**O**) Western blot analysis of Myh9, Dnmt1, and Brd4 protein in the hippocampus of WT and *Arrb2*^−/−^ mice. ns: *p* > 0.05, * *p* < 0.05, *** *p* < 0.001, unpaired two-tailed Student’s *t*-test, *n* = 6 per group.

We experimentally investigated whether *Arrb2* regulates the downstream genes as predicted by in silico analysis. First, as previously described [7], we used the CRISPR/Cas9 technique to knock out a 19-bp region of the first exon and the subsequent partial intronic region, totaling 350 bp of the *Arrb2* gene in C57BL/6 mice (Figure 7A). Compared with the wild-type (WT) group, the levels of *Arrb2* mRNA and protein in *Arrb2*^−/−^ mice were significantly reduced (Figure 7B,C). These results confirmed the successful construction of *Arrb2*^−/−^ mice. Since all eight strong candidate genes are expressed in the hippocampus, we next measured the relative mRNA expression levels of *Dnmt3a*, *Myh9*, *Ccdc88a*, *Dnmt1*, *Becn1*, *Gng2*, *Psmb6*, and *Brd4* in the hippocampus of WT and *Arrb2*^−/−^ mice using qRT-PCR (Figure 7D–K). We found that the mRNA expression levels of all genes in the *Arrb2*^−/−^ group showed reductions to varying degrees, with significant decreases observed in *Myh9*, *Dnmt1*, and *Brd4*. Western blot analyses further confirmed concordant protein-level reductions for these three targets (Figure 7L–O). Notably, functional analyses across human, mouse, and rat databases revealed associations between these three genes and the autism-related annotations.

### 3.9. Abnormal PKA-Induced Synapsin I Activation in the Hippocampus of Arrb2^−/−^ Mice

**Figure 8:**
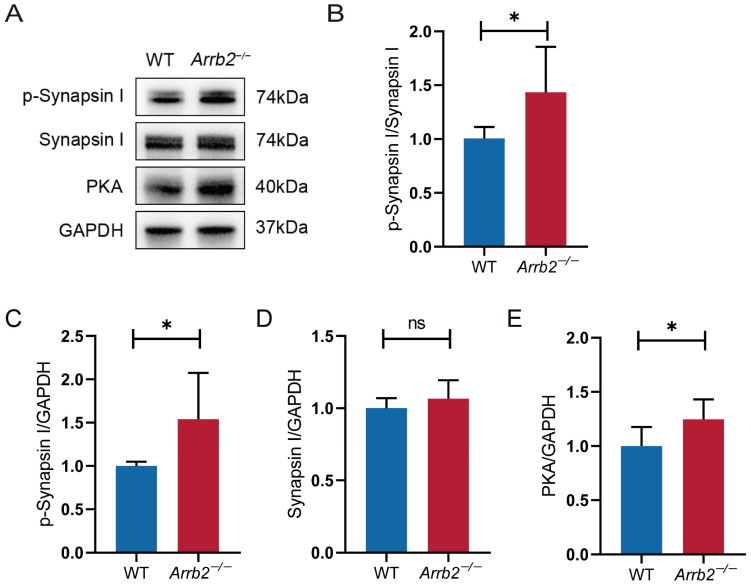
Effect of *Arrb2* knockout on PKA-induced synapsin I activation. (**A**) Representative Western blot images of the target proteins PKA, p-Synapsin I, Synapsin I, and the internal control proteins GAPDH in the hippocampus of WT and *Arrb2*^−/−^ mice. (**B**) Statistics of the relative expression of PKA in the hippocampus of the two groups of mice. (**C**–**E**) Statistics of the relative expression of p-Synapsin I and Synapsin I. ns: *p* > 0.05, * *p* < 0.05, unpaired two-tailed Student’s *t*-test, *n* = 6 per group.

Previous enrichment analysis of *Arrb2*-correlated genes identified abnormal synaptic transmission as the most enriched MPO in four brain regions: amygdala, cerebellum, hippocampus, and prefrontal cortex. It has been shown that Synapsin I regulates the binding of the actin cytoskeleton to synaptic vesicles via phosphorylation–dephosphorylation dynamics [68]. This process provides a molecular basis for synaptic vesicle cytosolic mobilization. Therefore, we measured p-Synapsin I (Ser9) levels in the hippocampus using Western blotting (Figure 8A). The p-Synapsin I/Synapsin I ratio was elevated in *Arrb2*^−/−^ mice compared to WT mice (Figure 8B–D), indicating Synapsin I overactivation. We further examined the upstream signaling pathway of Synapsin I [69] and observed a significant increase in PKA in *Arrb2*^−/−^ mice (Figure 8E). These findings suggest that PKA-induced aberrant Synapsin activation contributes to abnormal synaptic transmission resulting from *Arrb2* knockout.

## 4. Discussion

Employing systems genetics approaches in the BXD RI strains, we identified significant expression variability of *Arrb2* across four brain regions. Elevated *Arrb2* expression levels showed strong correlations with the severity of autism-related behavioral traits. This observation aligns with previous reports of de novo *Arrb2* mutations detected in peripheral blood samples from ASD patients [5]. However, ASD arises from complex gene–environment interactions, so further investigation into *Arrb2*’s mechanistic role in autism pathogenesis is essential. Through integrative functional analyses, we identified eight *Arrb2*-regulated genetic modulators that may cooperatively contribute to ASD development. Experimental validation in *Arrb2*^−/−^ mice confirmed significant downregulation of three modulators (*Myh9*, *Dnmt1*, and *Brd4*) and aberrant PKA-mediated Synapsin I activation in hippocampal tissues.

Traditional single-brain-region approaches may fail to comprehensively assess gene regulatory roles in complex behaviors. Through multi-omics integration across the amygdala, cerebellum, hippocampus, and prefrontal cortex, our findings demonstrate that increased *Arrb2* expression significantly correlates with core autism phenotypes, including learning stimulus extinction, social impairments, and anxiety. These findings establish *Arrb2* as a critical modulator of cross-regional neural circuitry. Notably, prepulse inhibition (PPI) of the acoustic startle shows a striking inverse correlation with *Arrb2* expression in the cerebellum, hippocampus, and prefrontal cortex. Mice with high *Arrb2* levels exhibit hypersensitivity to environmental stimuli and impaired suppression of startle responses, similar to the behavioral dysregulation observed in autistic individuals exposed to noisy environments. While previous studies showed that *Arrb2* knockout alleviates 5-HT1BR-induced PPI deficits [61], our study provides the first multi-regional evidence linking *Arrb2* expression to complex behavioral phenotypes. These results confirm that complex behaviors, such as learning, memory, emotion, and social interaction, are not independently completed by a single brain region. This highlights the necessity of studying multi-regional neural circuits and their signaling mechanisms when investigating autism and related neurodevelopmental disorders.

To investigate the biological functions of *Arrb2*, we performed functional enrichment analysis of *Arrb2*-coexpressed genes, which revealed four pathways (insulin signaling, glutamatergic synapses, dopaminergic synapses, and MAPK signaling) to be consistently enriched in the amygdala, cerebellum, hippocampus, and prefrontal cortex. Recent studies demonstrate that all these pathways are related to autism, supporting the importance of *Arrb2* in autism pathogenesis. Intranasal insulin [70] or subcutaneous IGF-2 administration [71] ameliorates social and behavioral deficits in autistic mouse models. Elevated GABA/glutamate ratios have emerged as a metabolic signature of mild autism and a potential diagnostic biomarker [72]. Furthermore, developmental dopamine signaling disruptions may drive aberrant neural circuitry and autistic-like behavioral phenotypes [73,74]. p38 MAPK signaling is known to regulate microglial activity and ASD [75]. Although *Arrb2* has been established in dopaminergic synapses and MAPK pathways, our study provides the first evidence linking it to insulin signaling and glutamatergic synaptic pathways. Notably, MAPK acts downstream of insulin signaling and dopaminergic synapses, collectively modulating cellular proliferation, differentiation, survival, and metabolism. Future research should investigate whether direct causal relationships exist between *Arrb2*, these four cross-regional signaling pathways, and ASD-related symptoms. Future research could further explore the direct causal relationship among the three, if any and whether *Arrb2* integrates cross-regional signaling to drive autism-related symptoms.

*Arrb2*-correlated genes expressed in multiple brain regions may participate in critical biological processes, contributing to brain-wide functional homeostasis. We constructed a molecular regulatory network using genes expressed in at least three brain regions that primarily or secondarily interact with *Arrb2*. Within our novel composite scoring system, a node degree > 1 served as the prerequisite for protein–protein interaction relevance to *Arrb2* and its correlated genes. The tissue-correlation analysis (*p* < 0.05) provided a stringent threshold to quantify the similarity of gene co-expression patterns across distinct cell types, tissues, or anatomical regions, effectively minimizing false-positive associations. The documented ASD associations of candidate genes in cross-species databases (human/mouse/rat) significantly enhanced their biological plausibility. Notably, ASD-associated differential expression patterns show conserved features between humans and mice. These cross-species parallels provide a critical bridge between animal models and clinical applications.

Through multi-tiered filtering, we identified eight strong candidate genes (*Dnmt3a*, *Myh9*, *Ccdc88a*, *Dnmt1*, *Becn1*, *Gng2*, *Psmb6*, *Brd4*), cumulatively representing 80% of the total scoring weight. These proteins participate in diverse biological processes, offering a multifaceted framework for understanding autism etiology. To explore the regulatory effect of *Arrb2* on downstream genes, we generated *Arrb2* knockout mice via CRISPR/Cas9. The experimental results showed significant downregulation of *Myh9*, *Dnmt1*, and *Brd4* mRNA levels in the hippocampus of *Arrb2*^−/−^ mice. This consistent change in expression patterns supports the hypothesis that *Arrb2* contributes to autism pathogenesis by regulating *Myh9*, *Dnmt1*, and *Brd4*, although differently in different tissues. Notably, these three genes were shown to be linked to autism-related terms in functional correlation studies (Table 2). *Dnmt3a* and *Dnmt1*, key DNA methyltransferases, mediate de novo methylation and methylation maintenance, respectively, critically regulating genes governing neuronal differentiation, migration, and circuit assembly. A previous genetic study found that intronic Single nucleotide polymorphisms (SNPs) in both genes show robust ASD associations [76]. Subsequently, multiple studies further corroborated that both underexpression [77] and overexpression [78] of *Dnmt1* disrupts transcriptional regulation in neurodevelopmental contexts. *Myh9* encodes the heavy chain of myosin IIA, coordinating actin filament dynamics and cytoskeletal remodeling through interactions with other myosin isoforms. Substantial evidence has shown that dysregulation of the actin cytoskeleton-related pathways that control dendritic structures may have a significant impact on the pathogenesis of autism [79,80,81]. *Brd4*, a bromodomain-containing transcriptional regulator, binds acetylated histones to modulate transcriptional programs. In 2012, Iossifov et al. first reported de novo *Brd4* deletions in ASD patients [82]. This variant led to a loss of function, preventing the increase in spine formation induced by *Brd4* in transfected neurons [83]. Subsequent studies have also discovered de novo missense variants, point mutations, or deletions of *Brd4* in ASD patients and developmental delay cases, correlating with neuropsychiatric phenotypes [84,85]. Furthermore, the remaining five candidate genes demonstrate multidimensional associations with neurodevelopmental disorders through distinct mechanisms as follows: epigenetic regulation (*Dnmt3a* [86]), cytoskeletal dynamics (*Ccdc88a* [87]), autophagy (*Becn1* [88]), proteostasis (*Psmb6* [89]), and transcriptional control (*Gng2* [90]). Our PheWAS analysis corroborated these pathway-specific associations. Although qRT-PCR revealed downward trends in mRNA levels for these genes, the changes did not reach statistical significance. This suggests either weaker regulatory effects of *Arrb2* on these genes or the presence of more complex regulatory networks worth further exploration.

Current clinical research has identified multiple ASD risk genes, such as *Shank3* [91], *Chd8* [92], and *Mecp2* [93]. However, due to its high heterogeneity, clinical diagnosis and treatment of ASD remain challenging. This study reveals the critical role of *Arrb2* in the pathogenesis of ASD by regulating the *Dnmt1*/*Myh9*/*Brd4* molecular network. By integrating cytoskeletal remodeling (*Myh9*), epigenetic dysregulation (*Dnmt1*), and chromatin remodeling/transcriptional regulation (*Brd4*) into the molecular framework of ASD, our findings provide novel insights for molecular subtyping and precision treatment strategies. Firstly, the expression levels of these molecules in peripheral tissues may serve as potential biomarkers for early genetic screening to indicate fetal ASD risk. Additionally, our findings provide a theoretical foundation for targeting these genes (*Myh9*, *Dnmt1*, and *Brd4*) as potential therapeutic candidates in ASD clinical interventions. For example, the downregulation of DNA methyltransferase *Dnmt1* may lead to dysregulation of epigenetic control of key neurodevelopmental genes, which is highly consistent with the abnormal DNA methylation patterns commonly observed in the brains of ASD patients [94]. This finding suggests that targeting epigenetic modifications (e.g., Methyltransferase inhibitors) could be a potential therapeutic strategy. Reduced expression of non-muscle myosin *Myh9* may disrupt cytoskeletal dynamics, affecting neuronal migration and synaptic formation, which is closely related to abnormal synaptic pruning and neural connectivity defects frequently observed in ASD patients. This result provides a theoretical basis for developing drugs targeting cytoskeletal regulation (e.g., RhoA/ROCK inhibitors) [95]. Although *Brd4* inhibitors, such as JQ1 are primarily applied in cancer therapy [96], recent studies suggest their novel therapeutic potential in neurological disorders. For instance, JQ1 rescues molecular and functional phenotypes of interneurons caused by *Mecp2* mutations in Rett syndrome [97]. It has been shown to modulate pathological mechanisms in amyotrophic lateral sclerosis (ALS) associated with *C9rof72* gene mutations [98]. In conclusion, the characteristics of this molecular network may help identify specific ASD subtypes and multi-target intervention strategies (e.g., simultaneously regulating epigenetic modifications and cytoskeletal stability), which may be more effective than single-target therapies.

There are still some limitations in our study. First, gene regulatory networks inherently involve complex non-linear processes, including feedback loops and spatiotemporal-specific regulation [99]. Despite employing multi-omics approaches to map *Arrb2*-associated pathways, current methodologies lack the resolution to dissect these delicate regulatory mechanisms. Second, while BXD mice provide genetic homogeneity and abundant datasets, they cannot fully replicate the multifactorial complexity and dynamic pathophysiological processes underlying human autism. Beyond genetic variations, potential compensatory mechanisms for epigenetic regulation or environmental factors (like prenatal stress or toxin exposure) may indirectly influence *Arrb2* expression and its downstream network. These gene–environment interactions still require validation in future human population cohorts. Third, our analysis was limited to hippocampal gene expression, and we should further assess brain-region specificity by profiling *Arrb2*-dependent regulation in the amygdala, cerebellum, and prefrontal cortex. Genetic epistasis (such as cooperative regulation between *Arrb2* and *Myh9*) may further modulate phenotypes, which will be explored in future conditional knockout models. Unraveling how these interactions orchestrate autism-associated neurobiological phenotypes will advance the understanding of ASD mechanisms.

## 5. Conclusions

In summary, by using the BXD RI strains as a genetic reference population, we identified a significant association between *Arrb2* and autism-related phenotypes across four brain regions: the amygdala, cerebellum, hippocampus, and prefrontal cortex. Further analysis confirmed cis-regulatory control of *Arrb2* expression. Through the construction of an *Arrb2*-correlated gene regulatory network, we screened out *Dnmt3a*, *Myh9*, *Ccdc88a*, *Dnmt1*, *Becn1*, *Gng2*, *Psmb6*, and *Brd4* as strong candidate downstream genes regulated by *Arrb2*. Subsequently, experimental validations in *Arrb2*^−/−^ mice verified the downregulation of *Myh9*, *Dnmt1*, and *Brd4* on mRNA and protein levels in the hippocampus, along with protein kinase A (PKA)-induced hyperactivation of Synapsin I. These findings lay the foundation for exploring the regulatory role of *Arrb2* in autism pathogenesis and its potential as a therapeutic target.

## Figures and Tables

**Table 1:** Primers used for qRT-PCR in hippocampal brain tissue.

Gene Name	Forward Primer (5′-3′)	Reverse Primer (5′-3′)
*Arrb2*	GAGGAACTCTGTGCGGCTTATCATC	GAGGAAGTGGCGTGTGGTTTCAG
*Dnmt3a*	CGCAAAGCCATCTACGAAGTCC	GCTTGTTCTGCACTTCCACAGC
*Myh9*	CACTACCAACCTCATGGAAGAGG	TCCAACTCCTGCCTCTGCTTCT
*Ccdc88a*	AAAGAGCGGCTCCTACACGATG	TCTAACCGAGCTTCTAAAGCAGC
*Dnmt1*	GGACAAGGAGAATGCCATGAAGC	TTACTCCGTCCAGTGCCACCAA
*Becn1*	CAGCCTCTGAAACTGGACACGA	CTCTCCTGAGTTAGCCTCTTCC
*Gng2*	GAAGCCAACATCGACAGGAT	GTTTTCTGAGGCTGGGACTG
*Psmb6*	GGCATGACCAAGGACGAATGTC	TTGGTCTCCCAAAAGCACCTGC
*Brd4*	GCCATCTACACTACGAGAGTTGG	ATTCGCTGGTGCTCTCCGACTC
*Gapdh*	AAGAAGGTGGTGAAGCAGG	GAAGGTGGAAGAGTGGGAGT

**Table 2:** Strong candidate genes regulated by *Arrb2*.

Gene Name	Parameters Considered for Scoring				
	Degree PPI Network	Tissue_Correlation *p* < 0.05 in Any One of Four Brain Regions (Count)	Autism Related	Differential in Any of the Three Comparisons	Total Score
*Dnmt3a*	4	3	Y	Y	5
*Myh9*	2	3	Y	Y	5
*Ccdc88a*	4	3	--	Y	4
*Dnmt1*	4	3	Y	--	4
*Becn1*	3	3	--	Y	4
*Gng2*	9	3	--	Y	4
*Psmb6*	5	3	--	Y	4
*Brd4*	3	3	Y	Y	5

In this table, “Y” stands for “Yes”, indicating that the gene is autism related or shows differential expression in any of the three comparisons. A double dash (--) signifies that the gene does not meet the above criteria.

## Data Availability

The expression data corresponding to BXD mice are available from our GeneNetwork portal (http://genenetwork.org/) with the accession #GN280, #GN56, #GN110, and #GN135. All other data presented are available either in the main manuscript or Supplementary Materials.

## Supplementary Materials

The following supporting information can be downloaded at: https://www.mdpi.com/article/10.3390/genes16050605/s1. Supplementary File S1: Arrb2 correlation with ASD phenotypes; Supplementary File S2: Arrb2 correlated genes across different brain regions; Supplementary File S3: Significantly enriched KEGG pathways and MPOs; Supplementary File S4: Details of 53 primary and secondary interactors of Arrb2; Supplementary File S5: Significantly differentially expressed genes between ASD and control samples.
